# The Human Genome Puzzle – the Role of Copy Number Variation in Somatic Mosaicism

**DOI:** 10.2174/138920210793176047

**Published:** 2010-09

**Authors:** Hasmik Mkrtchyan, Madeleine Gross, Sophie Hinreiner, Anna Polytiko, Marina Manvelyan, Kristin Mrasek, Nadezda Kosyakova, Elisabeth Ewers, Heike Nelle, Thomas Liehr, Samarth Bhatt, Karen Thoma, Erich Gebhart, Sylvia Wilhelm, Raimund Fahsold, Marianne Volleth, Anja Weise

**Affiliations:** 1 Jena University Hospital, Institute of Human Genetics and Anthropology, Jena, Germany; 2 Department of Genetics and Cytology, Yerevan State University, Yerevan, Armenia; 3 National Center of ‘Mother and Child’, Minsk, Belarus; 4 Research Center of Maternal and Children Health Protection, Department of Medical-Genetic Service, Yerevan, Armenia; 5 Institute of Human Genetics, Erlangen, Germany; 6 Biomedical Center, Homburg, Germany; 7 Middle German Praxis Group, Dresden, Germany; 8 Institute of Human Genetics, Magdeburg, Germany

**Keywords:** Copy number variations, pod-FISH, mosaicism.

## Abstract

The discovery of copy number variations (CNV) in the human genome opened new perspectives in the study of the genetic causes of inherited disorders and the etiology of common diseases. Differently patterned instances of somatic mosaicism in CNV regions have been shown to be present in monozygotic twins and throughout different tissues within an individual. A single-cell-level investigation of CNV in different human cell types led us to uncover mitotically derived genomic mosaicism, which is stable in different cell types of one individual. A unique study of immortalized B-lymphoblastoid cell lines obtained with 20 year interval from the same two subjects shows that mitotic changes in CNV regions may happen early during embryonic development and seem to occur only once, as levels of mosaicism remained stable. This finding has the potential to change our concept of dynamic human genome variation. We propose that further genomic studies should focus on the single-cell level, to understand better the etiology and physiology of aging and diseases mediated by somatic variations.

## COPY NUMBER VARIATION (CNV) OF HUMAN GENOME

Many forms of human genome variations are known and described including single-nucleotide polymorphisms, small insertion-deletion polymorphisms, variable numbers of repetitive sequences and genomic structural alterations. Recent developments in the genome-wide targeted technologies used to analyze structural variations have led to the identification of thousands of heritable copy number variations (CNV) occurring in both phenotypically normal and affected subjects [1]. These are submicroscopic CNV in DNA segments ranging from kilobases (kb) to megabases (Mb) in size and include deletions, insertions and duplications. However, they do not include those variants that arise from the insertion/deletion of transposable elements alone. About 24% of all CNV are associated with segmental duplications suggesting that non-allelic homologous recombination (NAHR) has been frequently involved in the genesis of these CNV [2]. Possibly, subsets of CNV not associated with segmental duplications may be formed or maintained by non-homology-based mutational mechanisms [3].

It is known that some CNV can influence gene expression and play a role in the etiology of common diseases such as diabetes, cancer, and heart conditions [4, 5]. Overall, 14.5% of genes in the OMIM morbid map overlap with CNV [6]. Interestingly, genes highly enriched in CNV are relevant to molecular-environmental interactions and influence response to specific environmental signals such as sensory perception of olfactory and chemical stimuli [7-10]. In addition, genes associated with disease susceptibility were found in regions exhibiting CNV. As example, Gonzalez [11] has shown that there are significant interindividual and interpopulation differences in the copy number of a segmental duplication encompassing the gene encoding CCL3L1, a potent HIV-1-suppressive chemokine and ligand for the HIV coreceptor CCR5. The investigation of HIV-1-positive and -negative individuals from groups with different geographical origins and ancestries (e.g. Africans or Europeans) revealed that a low *CCL3L1 *copy number was a major determinant for enhanced HIV susceptibility [11]. CNV have been also observed in other mammals such as mice [12] and chimpanzees [13]. 

## METHODS TO IDENTIFY CNV

Though about 6558 CNV are detailed in the Database of Genomic Variants (http://projects.tcag.ca/variation/) it is supposed that a certain number of CNV remains to be identified. To date, only genome-wide technologies have been available to detect such CNV and only DNA extracted from a multitude of cells could be analyzed by those approaches such as SNP arrays, whole-genome tiling-path array-comparative genomic hybridization (aCGH) platform, BAC-based aCGH and ROMA (= representative oligonucleotide microarray analysis) Acgh [1, 4-6]. However the detection frequency of *de novo* CNV strongly depends on the cut off criteria, especially when the size of an aberration is small. In conclusion, the confirmation of CNV *via *alternative techniques is particularly challenging. 

Recently, two single-cell-directed approaches were described as ‘parental-origin-determination fluorescence *in situ* hybridization’ (pod-FISH) [14] and ‘polymorphic deletion probe-based FISH’ (PDP-FISH) [15]. These techniques require CNV-region-specific bacterial artificial chromosomes (BAC; pod-FISH) and fosmid clones (PDP-FISH) to visualize copy number polymorphisms on homologous chromosomes. Pod-FISH is available for 225 CNV, based on specific BAC clones of more than 150 kb in length and with variation frequencies in populations of over 10%. The selected polymorphic regions represent size variations, detectable as different signal intensities with pod-FISH [14]. In contrast, PDP-FISH has been reported for three CNV loci using fosmid probes, which distinguish signal presence and absence rather than signal intensity differences [15].

## SOMATIC MOSAICISM OF CNV

For a long time it has been widely accepted that all cells in an individual are genetically identical, except for individuals with somatic mosaicism that causes disease, and for the rearrangements of the immunoglobulin and T-cell-receptor genes [16]. In contrast, more and more data are available demonstrating genomic variation in different tissues for numerical chromosome aneuploidy contributing to mosaicism as a global mechanism for example in germ cells, placenta, human brain, skin, liver and blood [17-20]. However, recent studies indicate that somatic mosaicism affecting known CNV might be seen as a rule rather than an exception. It has been suggested that sequence variation involving CNV between two normal subjects is higher than that for single-nucleotide polymorphisms [6]. The latter suggestions were confirmed as so-called putative *de novo* somatic CNV events in monozygotic (MZ) twins [21]. It is expected that MZ twins are genetically identical and that phenotypic differences between twins are mainly due to environmental factors. 

### Somatic CNV Mosaicism in Monozygotic Twins

Bruder [21] has investigated whether MZ twins display differences in CNV. The study of 19 pairs of MZ twins using the 32K BAC array platform revealed evidence for large scale CNV among them and suggests that these variations may be common, notably in somatic development. It was shown that the applied array platform was able to discover somatic mosaicism in 10-20% of nucleated blood cells. In one individual of the studied MZ twins two deletions in 4p and 11q, encompassing ~85 and ~22Mb, respectively, were found. Consultation of medical records revealed that this subject was diagnosed with chronic lymphatic leukemia (CLL) prior to sampling of his blood in the course of the study [22]. It remains to be assessed if the CNV were causative for the CLL. 

In addition to the concordant SNP genotypes, including several CNV that were shared by both twins of a pair, also a few discordances in A and B allele frequencies were found, suggesting putative *de novo* somatic CNV events. For instance, a CNV, which covers ~1.6 Mb on chromosome 2 and extends from SNP rs2304429 to rs1662987, implying a deletion, was found in one twin but not in the other. Two other methods (high-resolution melting curve analysis and pyrosequencing) have confirmed the deletion and indicated that it was present in approximately 70–80% of blood cells. The structural variations discovered in MZ twins suggest that somatic mosaicism for CNVs is relatively common in normal human cells [21]. 

### Somatic CNV Mosaicism within Humans

A recent study of different human tissues and organs from three subjects using a genome-wide tool (32K array) revealed the existence of somatic CNV mosaicism [22]. At least six CNV, affecting a single organ or one or more tissues of the same subject were observed. Cortex of the brain, pons and cortex of the cerebellum were obtained from three individuals and RP11-197P23 located in 1p36.33 showed variation only in experiments using the pons *vs* cerebellum of subject 3. Thus, it was suggested that somatic mosaicism for CNV occur in a substantial fraction of human cells.

### Somatic CNV Mosaicism within Mice

It has been shown that extensive *de novo* and recurrent CNV occur also *in vitro* in mouse embryonic stem cell lines derived from common parental lines, leading to mosaic animals containing variants of the zygote genome [23]. Clones with major chromosomal changes could not be transmitted into the mouse germ line and typically exhibited trisomies or multiple deletions or duplications. Some clones had a few small (1 to 2 Mb) CNV that did not affect germ-line transmission. More than half of these CNV appear to have arisen independently because they were observed in subclones isolated from different parental ES cell lines.

Overall, somatic CNV mosaicism patterns have not yet been fully resolved, because all previous studies were performed with whole-genomic DNA extracted from a large number of cells, approaching one million per assay.

## RECENT NEW INSIGHTS

As mentioned above PDP-FISH [15] and pod-FISH [14] provided recently the possibility to assess CNV variations *in situ*. The efficient use of both approaches in the clinical assessment of chimerism in hematopoietic stem cell transplantation, the exclusion of maternal contamination in prenatal diagnosis, the detection of uniparental disomy of single chromosomes, the determination of the origin of aberrant chromosomes, the proof of paternity by chromosomes and following single chromosomes in generations, was suggested and demonstrated [14, 15]. It is to emphasize that, in contrast to genome-wide approaches by pod-FISH it is possible to detect small deletions or duplications with a resolution of 150 kb and less on single cell level. Thus, this is a powerful approach for the discovery of adequately sized somatic mosaicism within a population of cells derived from one individual. 

### CNV Detection on a Single Cell Level in Clinical Cases

We have found not only interindividual differences, as expected, but also intraindividual differences determining the signal intensities of polymorphic BAC probes. Thus, in a woman with Turner syndrome and the mosaic karyotype 45,X,der(7)t(Y;7)(p11.1~11.2;p22.3)[122]/45,X[48], pod-FISH assessed the parental origin of the normal and derivative chromosomes 7 with two BACs (RP11-533E18 and RP11-45N9) of the 15 BACs tested [24]. Uniparental disomy 7 was excluded for both cell lines and the result was confirmed by microsatellite analysis. The pod-FISH results for this individual revealed the presence of different clonal cell lines within one tissue with respect to the investigated CNV [24].

### CNV Detection on a Single Cell Level in Leukemia

**Fig. (1).**
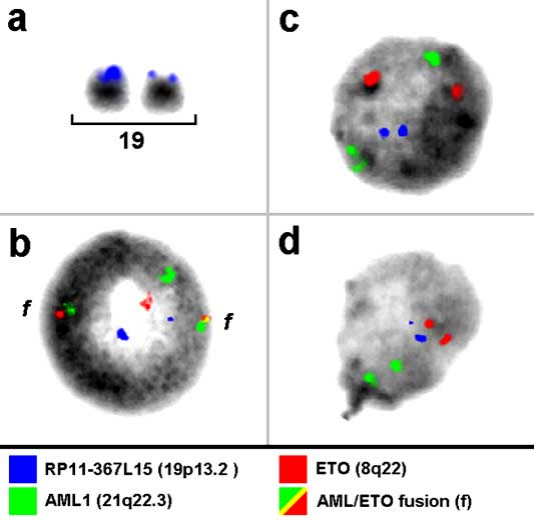
Single cell estimation of a copy number variation (CNV) by pod-FISH in an AML patient Different signal intensities for the CNV in 19p13.2 (RP11-367L15) are apparent on the two homologous chromosomes 19 in all metaphases of an AML patient with a t(8;21)(q22;q22.3) before bone-marrow transplantation.Interphase nucleus of the same AML-patient exhibiting the two fusion signals (f) typical for the t(8;21)(q22;q22.3).In the bone-marrow donor cells without the fusion signals for AML/ETO, 55% of the cells showed no signal intensity difference in the CNV region detected with RP11-367L15.However, the remaining 45% of the recipient cells showed signal intensity differences identical to those of the recipient.

pod-FISH [14] and PDP-FISH [15] have also been applied to the analysis of cellular chimerism after bone-marrow transplantation in leukemia patients. To identify a low level of chimerism, it is necessary to find polymorphic regions containing CNV in 100% of the transplantation recipient and donor cells. Using pod-FISH this condition was fulfilled for BAC RP11-367L15, mapping to chromosome 19p13.2, in a male suffering from *AML1–ETO*-positive acute myeloid leukemia (AML). After bone-marrow (BM) transplantation from a female donor, the cellular chimerism in BM was determined in 60% donor vs 40% recipient cells using a centromeric probe for the X chromosome. Surprisingly, no signal intensity variation for RP11-367L15 was found in 55% of the donor cells. The remaining 45% of donor cells showed the identical pod-FISH signal pattern as those of the recipient cells (see Fig. **1**).

### CNV Detection on a Single Cell Level in 3 Tissues of 10 Probands

**Fig. (2).**
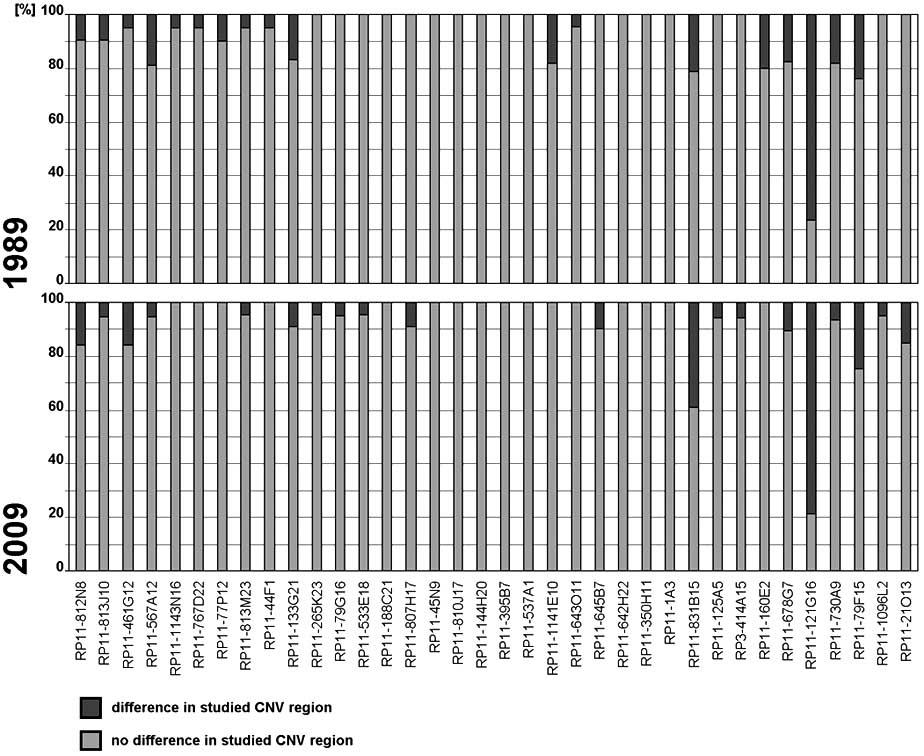
Results of pod-FISH analysis of two LCLs derived from one male subject established within a time interval of 20 years. The fraction of distinguishable cell lines by pod FISH remained practically the same for all 36 investigated CNV loci within the 20 year time interval.

Intraindividual differences detectable by pod-FISH turn out to be a common observation rather than an exception. To test whether different cell types have specific CNV patterns when studied at the single-cell level, we used chromosome-specific pod-FISH on metaphase spreads from 10 healthy individuals [16]. Three cell types were studied using 5 BACs with expected high population frequencies within CNV regions: T lymphocytes prepared from phytohemagglutinin (PHA)-stimulated peripheral blood; B-lymphocytes from Epstein–Barr-virus-immortalized B-lymphoblastoid cell lines (LCL) [25]; and fibroblasts from cultivated skin biopsy samples. In single cell types, we found cells with different and equal signal intensities for the same polymorphic BAC, varying between 0% and 95%, and random variations between all 10 individuals studied. Surprisingly, the variation ratio within the cells of one individual remained similar in all three cell types studied (*P* > 0.05). The results of this study suggest that somatic variation of CNV regions occurs in early embryogenesis [16]. To investigate whether once acquired CNV variations ratio remain stable through the whole life or undergo changes, we applied pod-FISH for 36 CNV on metaphase spreads of LCL from two individuals (subjects 1 and 2) established with a time interval of 20 years [16]. The age of the two probands was 25 and 30 years for the first sample, respectively. Cytogenetic analyses of all samples were performed using GTG banding and normal karyotypes were found [26, 27]. The experimental procedures performed on human tissue samples were approved by the Ethics Committee of Friedrich Schiller Jena University Hospital (internal code 1457-12/04). pod-FISH was done as previously reported [14]; the applied 36 BAC probes are listed in Fig. (**2**). Statistical analysis was performed using t-test (P=<0,01). As in previous studies also here we found two cell types with different signal intensity of the same BAC ranging from 0% to 100%. Interestingly, the variation ratio of BACs remained equal in LCL from different time points within a subject (Fig. **2**) and varied between the two individuals studied [16]. 

### Somatic CNV Mosaicism is a Common Finding

The mechanisms underlying the establishment of CNV mosaicism and their transmission through ‘mitosis remain unclear. Single-cell-CNV-focused approaches might only uncover a tip of the iceberg in the recently reported background of extensive chromosomal instability in human cleavage-stage embryos [28]. Several studies have predicted that some CNV and nonrecurring copy number variations in cancer cell lines, which are induced by aphidicolin or occur in some cases of Duchenne muscular dystrophy, Smith-Magenis syndrome or Pelizaeus–Merzbacher disease originate from nonhomologous end joining, fork stalling and template switching, or microhomology/microsatellite-induced replication mechanisms [29, 30]. Moreover, such evolutionarily and developmentally significant hotspots as fragile sites and aphidicolin-induced CNV might resemble human CNV [29].

## CONCLUSION

Overall, the presence of somatic CNV mosaicism in different individuals has been confirmed by several studies: Here we approach the problem of CNV mosaicism in different cell types and tissues of the same organism in different cell types and tissues of the same organism at differentially stages. The stable variation ratios of CNV detectable by pod-FISH have been shown to differ between individuals but not within healthy individuals. We suggest that the somatic recombination of polymorphic regions might occur at least at a relatively early time point in embryogenesis because all the well-differentiated cells studied have similar CNV mosaic patterns [16]. This hypothesis is substantiated by new findings of complex chromosomal imbalances involving not only whole chromosome abnormalities and uniparental disomies but also segmental deletions, duplications, and amplifications in human cleavage-stage *in vitro*-fertilized embryos [28]. Interestingly, when a CNV pattern is once established, the variation ratio seems to be stable throughout all tissues and remains similar during the whole life, as the data presented here underline.

Understanding these phenomena in more and finer details should open new perspectives in developmental physiology and in personalized medicine. The intraindividual specific mosaicism ratio at a certain disease susceptibility gene might have a higher impact than previously expected, especially for so-called ‘multi-factorial diseases’, and might also explain clinical genetic phenomena like diminished penetrance in autosomal dominant diseases or clinical signs without apparent mutations when only a single tissue is screened.
